# Design and Efficacy of Custom-Made Cleaning Splints for the Approximal Plaque Removal—A Laboratory Study

**DOI:** 10.3390/jcm13247763

**Published:** 2024-12-19

**Authors:** Stefan Rues, Theresa Becker, Valentin Bartha, Marcia Spindler, Sebastian Hetzler, Diana Wolff, Peter Rammelsberg, Andreas Zenthöfer

**Affiliations:** 1 Department of Prosthodontics, Medical Faculty, Heidelberg University, 69120 Heidelberg, Germanyandreas.zenthoefer@med.uni-heidelberg.de (A.Z.); 2 Department of Conservative Dentistry, Medical Faculty, Heidelberg University, 69120 Heidelberg, Germany; theresa.becker@stud.uni-heidelberg.de (T.B.); valentin.bartha@med.uni-heidelberg.de (V.B.); marcia.spindler@med.uni-heidelberg.de (M.S.); diana.wolff@med.uni-heidelberg.de (D.W.)

**Keywords:** splints, 3D-printing, plaque removal, approximal cleaning efficacy, interdental brushes

## Abstract

**Objectives:** Cleaning splints (CSs) can facilitate interdental brush (IDB) insertion and guide IDBs during cleaning movement. Cleaning efficacy with and without CSs was to be assessed and compared for a fully dentate (FD) and a partially edentulous (PE) situation. **Methods:** For two maxillary typodont models simulating either an FD situation or a PE situation with every second tooth missing, suitable IDBs were selected and each cleaning splint was designed and fabricated by 3D-printing. Before and after standardized cleaning, model teeth were photographed at three timepoints: (T1) clean surface, (T2) surface coated with artificial plaque, and (T3) after IDB cleaning. For each of the four test groups differing in dental status (FD, PE) and CS use (without, with), n = 10 tests/group were completed. After aligning corresponding photographs, pixel-based color difference ratios (T2-T3)/(T2-T1) were calculated. Approximal cleaning efficacy was set as the mean value over predefined regions of interest. Effects of CS use and dental status were analyzed with two-way ANOVA (α = 0.05). **Results:** CS use significantly improved mean approximal cleaning efficacy for the PE model (*p* = 0.001), whereas no difference was found with the FD model (*p* = 0.381). Improved cleaning efficacy with the PE model was only found in combination with a CS (factor combination model × CS use: *p* = 0.003). **Conclusions:** Cleaning splints might have the potential to improve interproximal hygiene and can be recommended for clinical use. Clinical studies should validate the results of this in vitro study and clarify if CSs could be beneficial for patients with restricted manual skills.

## 1. Introduction

Besides caries, periodontal disease is one of the major dental burdens and the sixth most prevalent chronic disease for patients [1]. It is desirable to preserve the patients’ natural teeth for the entire lifespan in a functional and esthetically pain-free condition. Adequate oral hygiene is a key factor, as a dysbiosis of the oral cavity favors plaque formation, leading to the described pathologies [2]. Furthermore, oral hygiene is also a prerequisite for the longevity of prosthetic restorations and preservation of teeth following periodontal therapy [3]. According to literature, regular mechanical removal of dental biofilm with additional use of dental floss or interdental brushes (IDB) can significantly reduce the risk for gingivitis and for plaque accumulation compared to solely regular toothbrushing [2,4,5,6]. However, in partially edentulous jaws, adjacent teeth are missing as well as their guiding function during IDB cleaning. The same is apparent or even aggravated if these patients are fitted with removable dental prostheses (RDP) [7]; however, the RDP per se does not lead to periodontal destruction [8]. Oral hygiene of proximal teeth or implant surfaces is hindered or complicated.

For this reason, it is necessary to improve oral hygiene at home and to optimize mechanical biofilm removal with additional aids. This requires appropriate oral hygiene instruction and motivation of the patients by dental professionals. During oral hygiene instruction, it is important to ensure that the appropriate aids are individually adapted to the anatomical conditions, for example, the size of the interdental brush to the interdental spaces to be cleaned [4]. A major drawback of using IDBs is their potential to traumatize the interdental soft tissues when handling IDBs [9], for example, because the tip has sharp edges and the circular shape makes it difficult to penetrate the interdental space in the correct direction. Especially in the molar region, the circular shape and the position of the proximal contact pushes the sharp-edged tip of the interdental space brush into the palatal margin of the gingiva when inserted from the buccal side. In this case, the circular morphology leads to an overlapping of the individual filaments during penetration into the interdental space with the consequence that the increasingly thicker diameter of the interdental brush becomes incompressible. This phenomenon is known as the clotting effect, which increases the risk of incorrect insertion of the interdental brush and traumatization of the gingival soft tissue [10]. In such cases in particular, a cleaning splint can help with interdental cleaning using interdental brushes by creating new guide surfaces [11].

Going back to prosthetic dentistry, tooth loss has become less progredient in recent decades but is still a burden, especially crossed with older age [12]. Thus, missing teeth are commonly recovered by double crown-retained and cast clasp-retained RDPs in partially edentulous jaws if multiple strategic implantations to enable fixed dental prostheses are no option. In order to create an adequate distribution of abutment teeth with a large supporting field, implants are also preferred nowadays. Although a prosthetic restoration with implants has numerous advantages, it should be borne in mind that one in five implants is diagnosed with peri-implantitis after 10 years. A case series reported a good clinical performance using individual cleaning splints [13]. To optimize the idea of cleaning splint-supported IDB use, a semi-automatically designed 3D-printed cleaning splint with ideal guide surfaces for the insertion of IDBs was developed. No information of the cleaning efficacy in use of these splints is available. The null hypotheses of this laboratory study were that neither dentition, IDB type, nor use of a cleaning splint will affect approximal interdental cleaning efficacy.

## 2. Material and Methods

This in vitro study aimed to identify a possible improvement in IDB cleaning efficacy with the use of cleaning splints (CSs) and to verify—prior to clinical use—that no negative aspects regarding dental hygiene come along with CS use. Cleaning efficacy was measured by average relative artificial plaque removal within regions of interest defined at proximal tooth surfaces. Two patient situations, (1) a fully dentate (FD) model and (2) a partially edentulous (PE) model, were used in this investigation representing the best and the worst case situation for IDB cleaning, respectively. Together with two cleaning protocols, (1) IDB cleaning without CSs and (2) IDB cleaning with CSs, there was a total of four study groups.

### 2.1. Typodont Models and IDB Selection

**Figure 1:**
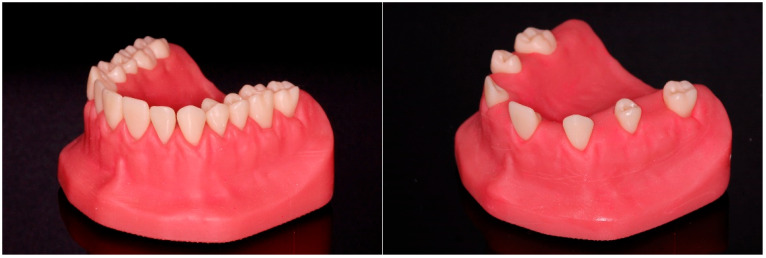
Typodont with regular interdental spaces (FD, **left**) and edentulous spaces (PE, **right**).

The teeth, gingiva mask, and model base of a commercial maxillary typodont model giving the situation of a periodontally diseased patient (model: A-GZ, gingival mask: A-GZ-WOK; Frasaco, Tettnang, Germany), were reverse-engineered (Geomagic Design X, 3D Systems, Rock Hill, SC, USA). Besides the FD model with regular interdental spaces, a partially edentulous (PE) model was designed as well by deleting every second tooth in the digital data set and closing the respective holes in the gingival mask. Using digital light processing (DLP; Max UV 385, Asiga, Alexandria, NSW, Australia), a model base (Freeprint Model grey UV, Detax, Ettlingen, Germany), a gingival mask (Freeprint Gingiva UV, Detax, Ettlingen, Germany), and test teeth with a basal core hole (V-Print Model, VOCO, Cuxhaven, Germany) were 3D-printed for each design and postprocessed according to the manufacturers’ instruction. Approximal tooth surfaces, which were of importance later on, were slightly polished. A thread cutter was used with the core hole enabling the fixation of the printed teeth in the model base with screws. The two assembled models based on 3D-printed components are shown in Figure 1.

Since cleaning efficacy strongly depends on the optimal choice of IDB size for the respective interdental space [14], an experienced dentist selected the IDB brush sizes (Curaprox, Curaden, Kriens, Switzerland) to be used in each interdental space of the FD model. For the CSs of the PE model, identical CS opening diameters were used as given for the FD model. Small CS openings were associated with a 1.0 mm guide surface offset and an IDB with 0.7 mm wire diameter, whereas large CS openings were associated with a 2.0 mm guide surface offset and an IDB with 1.7 mm wire diameter (Table 1).

### 2.2. Design and Fabrication of Cleaning Splints

**Figure 2:**
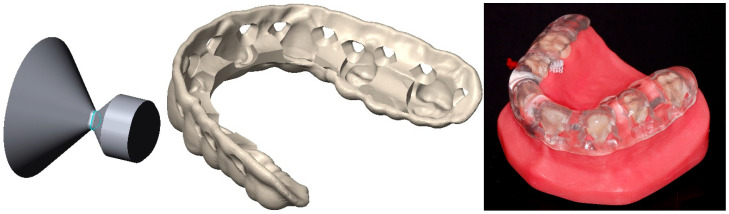
Punching die used to create triangular opening (**left**), final splint design for the PE model with IDB openings cut into the basic splint design and additional guide surfaces (**center**), and 3D-printed cleaning splint on applied to the PE model (**right**).

Based on laboratory 3D scans (D2000, 3Shape; Copenhagen, Denmark) of the two typodonts, basic splints were designed with a dental design software (Ceramill Mind Version 3.1, Ammann Girrbach, Pforzheim, Germany) ending above the prosthetic equator on the oral side. Wall thicknesses amounted to 3 mm on the buccal and 1.5 mm on the occlusal aspects. Smoothing was at maximum to keep interdental spaces free of splint material. The designed basic splints were exported together with the respective model scans in the design coordinate system (vertical axis = insertion direction of the splint). After importing the splint and model in CAD software (Geomagic Design X 2023.2.0, 3D Systems), individual IDB insertion axes were defined by connecting the respective highest points of the papillae on the buccal and oral side. These axes intersected with the interior surface of the splint. Punching dies with reference points 1.7 mm on the oral side of the narrowest cross-section were moved to that position and rotated (around the reference point) such that the punching die axis was identical to the projection of the IDB axis into the xy plane. Thus, all punching dies were oriented horizontally and their narrowest cross-section located 1.7 mm from the oral splint surface. Two sizes of punching dies with triangular cross-sections (Figure 2, left) were used: (1) a punching die with an opening size (diameter of the incircle of the narrowest cross-section) of 1.5 mm for IDB with wire diameters up to 1.3 mm and (2) a punching die with an opening size of 2.5 mm. IDB guide openings were cut into the splints by logically subtracting the punching dies from the basic splint. Finally, in the case of missing approximal teeth, IDB guide surfaces were created either 1.5 mm or 2.5 mm from the respective approximal tooth surface depending on IDB size (Table 1). For the PE model, the CS design (Figure 2, center), as well as the fabricated CS seated on the model (Figure 2, right), is displayed.

Final cleaning splint designs were 3D-printed using a DLP printer (Max UV, Asiga) and appropriate resin (Freeprint Splint 2.0, Detax). The finished designs for the cleaning rails were 3D-printed using a DLP printer (Max UV, Asiga, Alexandria, NSW, Australia) and the corresponding plastic (Freeprint Splint 2.0, Detax, Ettlingen, Germany). Completed cleaning splints were postprocessed according to the manufacturer’s instructions including ultrasonic cleaning with isopropanol for 2 × 3 min and final light curing with 2 × 2000 flashes (Otoflash, NK-Optik, Baierbrunn, Germany).

### 2.3. Image Aquisition and Evaluation of Cleaning Efficacy

**Figure 3:**
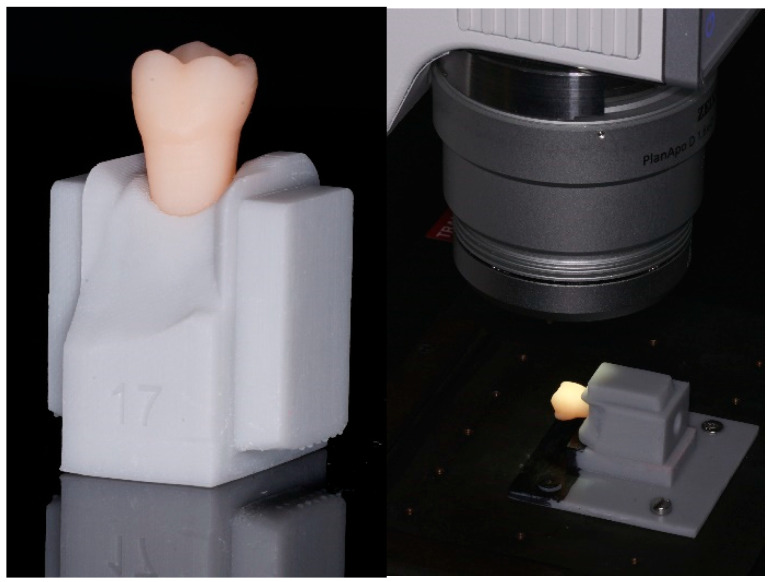
Individual sample holder for tooth 17 (**left**) and image-taking of the approximal surface with a digital microscope (**right**).

A base plate with a rectangular socket was fabricated by 3D-printing (Freeprint model grey, Detax) and screwed to the microscope table. For each tooth, a sample holder (Figure 3, left) with rectangular plugs fitting into the socket of the base plate was created to enable exact sample repositioning (Figure 3, right) when taking images with a digital microscope (Smartzoom 5, Carl Zeiss, Jena, Germany). In administrator mode, a job was created with fixed settings (lighting, magnification, table position, exposure time) for each approximal tooth surface. In the following, the job was executed in the user mode any time new images of the tooth surfaces were needed.

**Figure 4:**
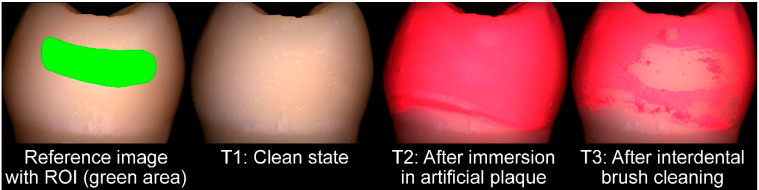
Exemplary images of the mesial side of tooth 17 for the clean state (T1), after artificial plaque application (T2), and after interdental brush cleaning (T3).

With each model, each n = 10 IDB cleaning procedures were carried out alternately with or without CSs. Before each IDB cleaning procedure, teeth were removed from the respective model and completely cleaned, firstly with ethanol and subsequently with steam. Images of the clean tooth surfaces (T1) were assessed before application of artificial plaque (Artificial Plaque, Nissin Dental Products, Kyoto, Japan). After air drying, the crowns of the teeth were immersed 3 times in artificial plaque for 20 s and removed from the artificial plaque liquid for another 20 s. Before image-taking of the situation with artificial plaque (T2), a drying time of at least 5 min elapsed. Then, teeth were applied to the respective model base, and IDB cleaning took place. After IDB insertion (new IDBs were used for each cleaning test), interdental spaces were cleaned by 3 forward-and-backward motion cycles. After removing the teeth from the model again, images of their state after IDB cleaning were taken (T3). The region of interest (ROI) for later evaluation of IDB cleaning efficacy was limited to the approximal surface area not accessible to regular tooth brushing. Further ROI limitations were the approximal tooth contact in a coronal direction and the gingival mask in an apical direction, since the possibility could not be excluded that artificial plaque might have been removed in subgingival areas when taking the teeth from the model. An exemplary image sequence (T1–T3) as well as the reference image with the ROI marked in green is given in Figure 4.

**Figure 5:**
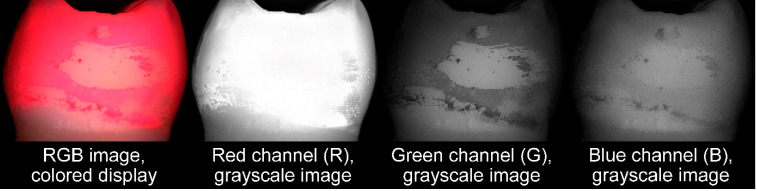
RGB channels for the exemplary image of the mesial side of tooth 17. It can be seen that the best contrast between clean tooth surface and surface areas covered with artificial plaque are given by the green channel.

**Figure 6:**
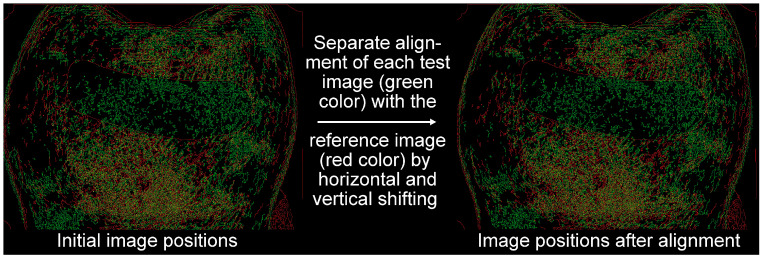
Overlaid edge detection images of the reference image and a test image, exemplarily shown for the mesial surface of tooth 17. Small deviations in tooth position (**left side**) when taking the reference image (red color) and the actual test image (green color) were corrected by manual alignment (**right side**).

**Figure 7:**
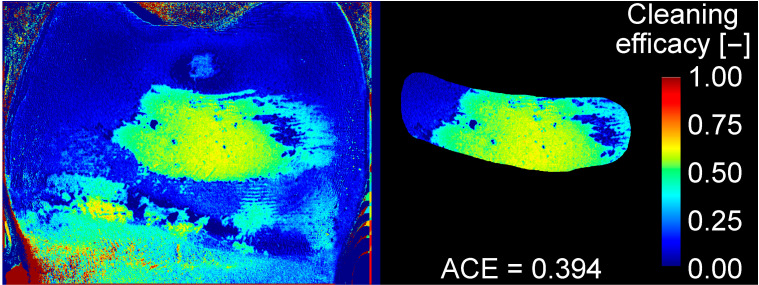
IDB cleaning efficacy calculated for the example shown in Figure 4, both for the complete tooth (**left**) and the region of interest (ROI, **right**). Cleaning efficacy ranges between values 0 (no cleaning) and 1 (perfect cleaning). For every cleaned surface, the mean approximal cleaning efficacy (ACE) within the ROI was calculated.

As can be seen from Figure 5, the red and blue channels of the RGB image (by superposition of three primary colors (R: red, G: green, B: blue), any color can be displayed with respective intensities of the three color channels) of an IDB-cleaned tooth were not suitable to show differences between cleaned and stained regions, whereas in the greyscale image of the green channel, this differentiation could be well performed. Because of this, only the green channel of the RGB images was used to assess IDB cleaning efficacy. Since repositioning of the teeth for image taking was not perfect, small deviations in position were corrected by manually aligning images (Figure 6) for each of the 3 states (T1–T3) with the respective reference image (containing the ROI definition) using contours given by edge detection (Matlab R2022a, Mathworks, Natick, MA, USA). Cleaning efficacy was calculated for each pixel of the image (Figure 7, left) as the quotient of the greyscale value difference T2–T3 (plaque removal due to IDB cleaning) and the grayscale difference T2–T1 (maximum difference, states with/without artificial plaque):

$$cleaning\;efficacy=\frac{T2-T3}{T2-T1}$$

In the case of calculated cleaning efficacy <0 or cleaning efficacy >1, cleaning efficacy was set to values of either 0 (no cleaning) or 1 (perfect cleaning), respectively. For this study, only the values inside the ROI were considered (Figure 7, right), and the mean value (APE: approximal cleaning efficacy) was saved for statistical evaluation.

### 2.4. Statistical Analysis

Before beginning statistical evaluation with SPSS Ver. 28 (IBM, Amonk, NY, USA), averaged cleaning efficacy values for each test were calculated based on the respective ACE values of tooth surfaces cleaned with the identical IDB type. Since data for cleaning efficacy were not homoscedastic, ranked data (which were homoscedastic and normally distributed according to the Levene and Shapiro–Wilk tests) were used for ANOVA (three factors: model, IDB type, use of CSs; level of significance: α = 0.05) and post hoc Tukey’s tests. For additional pairwise comparisons, *t*-tests were carried out.

## 3. Results

**Figure 8:**
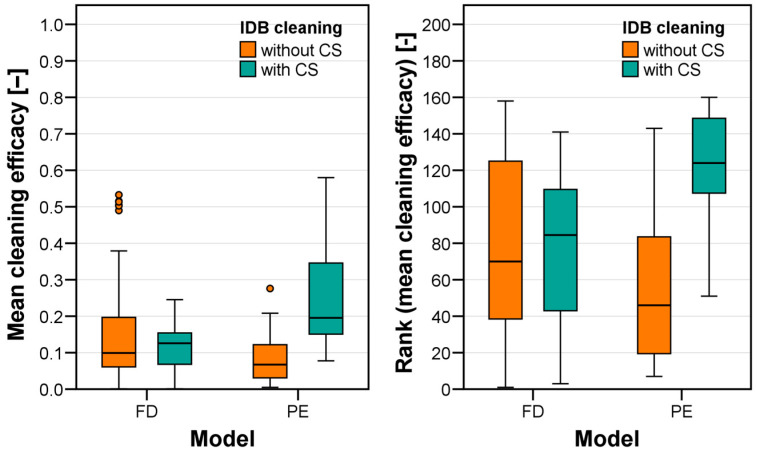
Mean cleaning efficacy (**left**, ACE values of each test averaged for approximal surfaces cleaned with identical IDBs) and associated ranks (**right**) for test groups differing in dentition (FD: fully dentate model, PE: partially edentulous model) and use of CSs during IDB cleaning.

The measured cleaning efficacy was first visualized (Figure 8) solely for groups differing in factor models (FD vs. PE) and use of CSs (without CSs vs. with CSs). Cleaning efficacy was similar for all test groups but the PE model cleaned with CSs (FD without CSs: 0.153 ± 0.147, rank 78 ± 51; FD with CSs: 0.118 ± 0.062, rank 78 ± 39; PE without CSs: 0.082 ± 0.069, rank 53 ± 40; PE with CSs: 0.250 ± 0.142, rank 122 ± 31). ANOVA (based on the ranked data, Table 2) indicated that the factor CS use as well as the factor combination model × CS use significantly affected mean approximal cleaning efficacy. This was the case because use of CSs did only lead to an increased cleaning efficacy for the PE model (*t*-test: *p* < 0.001) but not for the FD model (*t*-test: *p* = 0.088).

**Figure 9:**
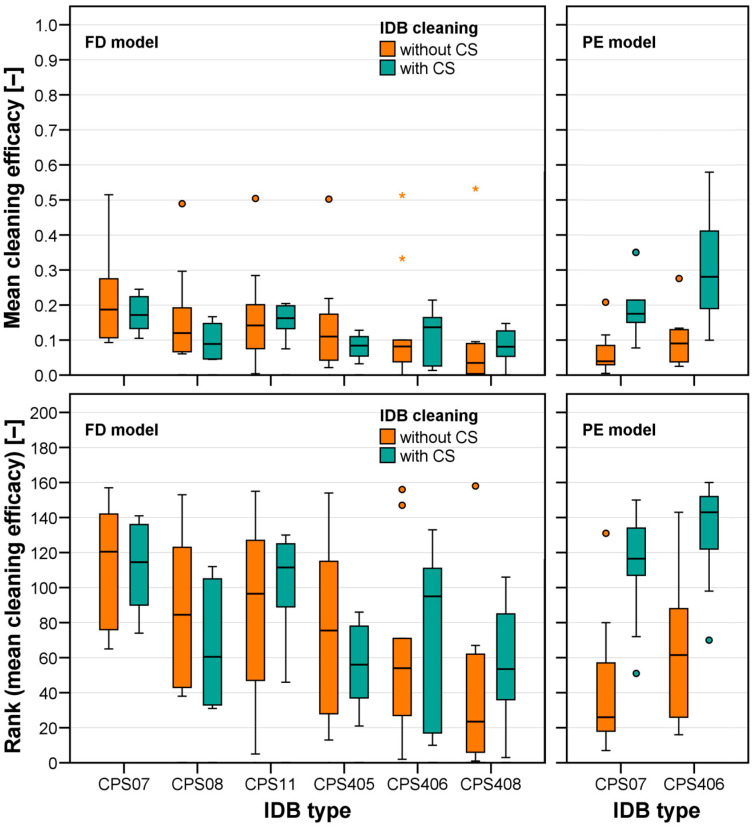
Mean cleaning efficacy (**top**, ACE values of each test averaged for approximal surfaces cleaned with identical IDBs) and associated ranks (**bottom**) for test groups differing in dentition (FD: fully dentate model, PE: partially edentulous model), IDB type, and use of CSs during IDB cleaning. Circles state moderate outliers, whereas asterixes state severe outliers.

When having a closer look and differentiating also approximal surfaces cleaned with identical IDB types (Figure 9), it was found that this factor as well as the factor combination model × IDB type significantly influenced cleaning efficacy (*p* = 0.001). The best cleaning efficacy was found for the FD model with small IDBs (CPS 07, CPS 08, CPS 11), whereas the performance of the larger IDB (CPS406) was better than that of the smaller one (CPS07) with the PE model.

**Figure 10:**
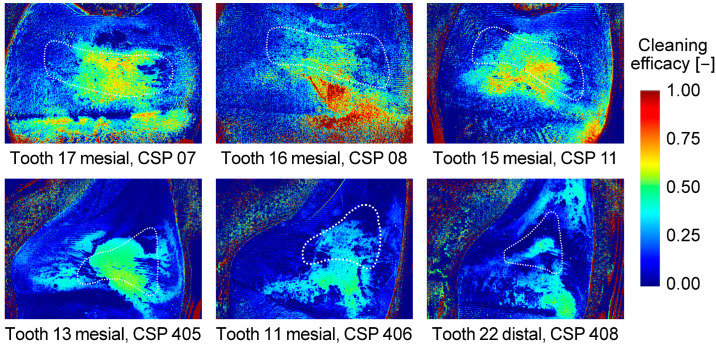
Representative cleaning results gained with the use of different IDBs for the FD model with CSs. The dotted white line indicates the respective ROI.

**Figure 11:**
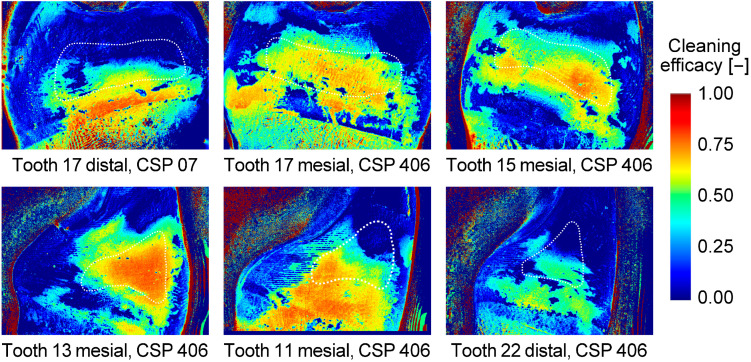
Representative cleaning results gained exemplarily with the use of different IDBs for the PE model with CSs. The dotted white line indicates the respective ROI.

Representative color plots showing the cleaning efficacy with CSs use are given for the FD model in Figure 10 and for the PE model in Figure 11. Cleaning efficacy was best at the narrowest part of the interdental space. The more curved the approximal tooth surfaces, the less efficient was the ACE next to the narrowest part in the FD model. This effect was drastically reduced in the PE model with guide surfaces paralleling the approximal tooth surface. Cleaning efficacy was best at the gingival level (vertical position of the funnel-shaped opening) and became worse for the coronal aspects of the approximal surfaces.

## 4. Discussion

The study hypotheses had to be partially rejected. Whilst mean cleaning efficacy was comparable in both test groups with the FD model, the PE situation yielded better mean cleaning efficacy when a CS was used. In addition, significant differences in mean cleaning efficacy were observed for approximal tooth surfaces cleaned with different IDB types.

With regard to IDB types, it has to be considered that the IDB sizes chosen for the FD model continually increased from posterior to anterior interdental spaces. In the clinical context, it can be mentioned that IDBs have been shown to offer additional benefits in reducing plaque beyond tooth brushing only, particularly in wider interdental spaces [6]. Within this study, the pattern of smaller and wider interdental spaces was just a coincidence with the copied typodont model. For clinical IDB selection, patterns will differ from patient to patient. Since tooth geometry (e.g., curvature) and interdental space geometry differ tremendously between the posterior and anterior regions and the influence of these parameters has been shown [15], the effect of IDB type seen here might have been influenced by these other factors. As can be seen in Figure 10 depicting the results of the FD model, ACE was better on the rather flat approximal surfaces of a molar compared to the convex approximal surfaces of anterior teeth with a high curvature. For the latter case, high cleaning efficacy could only be seen at the narrowest part of the interdental space. For the PE model, ACE with CS use (Figure 11) was much improved. This was most likely caused by the change in “interdental space” geometry with the artificial guide surface running in parallel to the approximal tooth surface at a given distance slightly above the wire diameter. It should be considered, however, that in this study only one standardized bristle design was used across all IDBs, which may not totally address the potential impact of variations in bristle shape or stiffness on cleaning efficacy as shown by Langa et al. in context of tooth brushes [16]. From a clinical view, especially in periodontitis patients or those with complex double crown-retained or implant-retained removable prostheses, adjacent teeth and implant abutment surfaces in particular become plaque predilection sites that are difficult to clean. Respective anatomical issues can be anticipated by the parallel guide surfaces of CSs. Inadequate hygiene of interdental spaces may lead to caries, periodontal disease, or periimplantitis [17,18,19]. From a case series of a manually fabricated individual cleaning splints, beneficial effects on cleaning efficacy can be assumed in PE situations [13]. The presently tested further development of those cleaning splints and resulting cleaning efficacy are thus in on line with these observations. Cleaning splints that are simple to fabricate and equipped with guide surfaces for interdental brushes may support the daily cleaning of difficultly to reach tooth or implant surfaces. Going back to the in vitro study, a large number of standardizations were used in this study in order to achieve identical conditions in the test groups and the greatest possible clinical relevance.

Standardized photographs were taken with a digital microscope, using the same focus, light, and magnification each time. In order to control for the initial state of the tooth surfaces and after re-cleaning, baseline photographs were taken as comparators. Software for evaluation of the proportion of cleaned and un-cleaned areas was compiled to automatize and homogenize the amount of residual plaque.

The majority of published studies on the cleaning efficacy of teeth convert color images after artificial plaque application (T2 in our tests) and after tooth brushing or IDB cleaning (T3 in our tests) to black–white images. In consequence, there are only 2 distinguishable states at each pixel of the image: (1) no plaque or (2) plaque. Such results are dependent on the threshold chosen for black–white conversion. The advantage of our approach is that there is a continuous measure for ACE between 0 (no cleaning) and 1 (completely clean), but an additional image of the clean state (T1 in our tests) is required. With the common method, images after plaque application are not mandatory if the whole tooth is coated, enabling measurements based on a single image. In contrast, the images taken at T1–T3 in this study had to be aligned before evaluation to correct small deviations in sample repositioning despite the best possible standardization. Similar to the planimetric evaluations often executed with tooth cleaning [20], predefined ROIs were defined with the reference images used for image alignment. A limitation of this study was that subgingival tooth surfaces had to be excluded from evaluation, since it could not have been ruled out that (additional) plaque was removed by contact with the gingival mask during tooth removal from the model after IDB cleaning. Furthermore, even if the gingival mask consisted of a resilient resin, it behaved more stiffly compared to a real gingiva. Subgingival plaque removal can, therefore, only be assessed with certainty in a clinical study.

Some different artificial plaque agents are used in in vitro studies. White-colored titanium oxide (which is used in commercial scan sprays) provides a good contrast on black typodont teeth [21] or, vice versa, black dye can be used on white teeth [20,22]. In order to assess plaque removal, some studies use off-label or dental detergents such as green occlusion spray [15,23] or blue marker dye [10]. The red-colored artificial plaque made by Nissin Dental Products Inc. [14,24,25] was deemed by the authors to mimic the properties of real dental plaque best and was chosen for our in vitro experiments. The advantage of colored artificial plaque agents is that they are suitable not only for use with typodont teeth but also for use with natural teeth. In this study, the standardized cleaning cycle was chosen to be short to avoid a potential influence of brushing duration and to focus on the potential effects of the splint. However, Kim et al. also demonstrated that longer brushing durations may further enhance cleaning efficacy [14]. Future studies should further investigate the effect of varying brushing duration in context of cleaning splints.

To this end and as an outlook, CSs are not expected to cause harmful effects in a clinical application. This recent translational study showed a comparable average ACE in FD situations and positive effects for PE when CSs were used. Since this could be confirmed, the next planned step is a clinical trial. Here, additional factors, such as motor skill impairment of a patient, could be assessed for cleaning efficacy with CSs, which was not possible in a laboratory setting. Use of CSs could also be combined with other aids such as CAM-fabricated individual brush handles for patients with limited manual dexterity [26]. As demonstrated in a pilot study by Pieper et al. [27], use of robot assistants may be a feasible option to support dental hygiene in patients with impaired motor skills or vision. The aim of the investigated cleaning splint was to increase plaque removal efficiency. In this context, it should be mentioned that other innovative cleaning aid designs, such the use of an Archimedean screw design in interdental rubber picks, have been shown to be beneficial in improving cleaning efficacy. Moreover, they were able to reduce the force required for cleaning [28]. However, the level of evidence for the effect of rubber bristles in reduction of gingivitis remains rather weak [29]. Furthermore, the level of evidence supporting IDB clinical effectiveness compared to tooth brushing alone was considered to be rather weak, highlighting the need for further clinical studies [30] as well as the need for further studies to evaluate alternative interdental cleaning tools that might fit for specific anatomical situations. Several studies have compared the efficiency of IDBs to dental floss. Worthington et al. assessed the efficacy of various interdental cleaning devices, including IDBs and dental floss in addition to toothbrushing [2]. They found a superiority of IDBs compared to floss with a low certainty that IDBs reduce gingivitis more effectively compared to floss at one and three months, and the evidence for plaque reduction was inconsistent. However, a recently published RCT found a significant beneficial effect of IDBs in the reduction of periimplant mucositis compared to dental floss [31]. Another recent study reported that the concept of increasing oral hygiene mainly refers to quantitative aspects such as brushing duration. This was not reflected in an increased measurable leaning efficacy [32]. Both the use of cleaning splints and the comparative investigation of different cleaning aids might address the qualitative aspect of cleaning.

As limitations of this laboratory study, the investigation of only one standardized brushing cycles should be particularly considered. Future studies should include a wider range of brushing durations. Moreover, only IDBs were investigated, meaning that further studies could also compare the effectiveness of different cleaning aid designs with the investigated splint.

## 5. Conclusions

Within the limitations of the laboratory study, use of cleaning splints to assist interdental brush cleaning showed no negative impact on the mean approximal cleaning efficiency. Moreover, in partially edentulous with missing guide surfaces situations its use was more effective. This supports the idea that patients with manual impairments or specific anatomical tooth restrictions difficult to clean, might benefit in particular from use of cleaning splints in the sense of improvements of interdental hygiene. Clinical studies should clarify if these assumptions as well as preclinical findings can be confirmed.

## Figures and Tables

**Table 1:** Interdental brushes selected for use with the FD and the PE model. CSs were provided with funnel-shaped openings with triangular cross-sections. Opening size (diameter of the incircle to the triangular cross-section) at the narrowest part was either 1.5 mm or 2.5 mm depending on the IDB size. For the PE model, guide surfaces with an offset distance of either 1.0 mm or 2.0 mm from the respective tooth surfaces were added to the CSs.

Model	Interdental Space or Tooth Surface *	IDB Type	IDB Wire Diameter/Brush Diameter	CS Opening Size/Guide Surface Offset
FD	17 d, 17/16, 26/27, 27 d	CPS 07	0.7 mm/2.5 mm	1.5 mm/-
	16/15, 24/25, 25/26	CPS 08	0.8 mm/3.2 mm	1.5 mm/-
	15/14, 14/13, 23/24	CPS 11	1.1 mm/5.0 mm	1.5 mm/-
	13/12, 12/11, 21/22	CPS 405	1.3 mm/5.0 mm	1.5 mm/-
	11/21	CPS 406	1.7 mm/6.5 mm	2.5 mm/-
	22/23	CPS 408	2.2 mm/8.0 mm	2.5 mm/-
PE	17 d, 26 d	CPS 07	0.7 mm/2.5 mm	1.5 mm/1.0 mm
	17 m, 15 m/d, 13 m/d, 11 m/d, 22 m/d, 24 m/d, 26 m	CPS 406	1.7 mm/6.5 mm	2.5 mm/2.0 mm

* m: mesial tooth surface/d: distal tooth surface.

**Table 2:** Results of the ANOVA based on ranks of the measured cleaning efficacy.

Factor (Combination)	F	Significance
Model	0.057	*p* = 0.811
CS use	15.395	*p* < 0.001
IDB type	4.375	*p* = 0.001
Model × CS use	13.362	*p* < 0.001
Model × IDB type	13.776	*p* < 0.001
CS use × IDB type	0.849	*p* = 0.518
Model × CS use × IDB type	0.176	*p* = 0.676

## Data Availability

The data presented in this study are available on justified personal request from the corresponding author. The data are not publicly available in order to avoid unauthorized double utilization.
